# Atrial Fibrillation Ablation Technology Adoption and Procedural Metrics: An 11-Year Longitudinal Service Evaluation at a UK Tertiary Centre

**DOI:** 10.3390/jcm15176894

**Published:** 2026-09-06

**Authors:** Ibrahim Antoun, Ahmed Abdelrazik, Thet Su Su, Sharon H. W. Man, Mahmoud Eldesouky, Kaung Myat Thu, Zakariyya Vali, Mokhtar Ibrahim, Riyaz Somani, G. André Ng

**Affiliations:** 1 Department of Cardiology, University Hospitals of Leicester NHS Trust, Glenfield Hospital, Leicester LE3 9QP, UK; 2 Department of Cardiovascular Sciences, Clinical Science Wing, Glenfield Hospital, University of Leicester, Leicester LE3 9QP, UK; 3 Department of Cardiology, University Hospitals of Plymouth NHS Trust, Plymouth PL6 8DH, UK; 4 National Institute for Health Research Leicester Research Biomedical Centre, Leicester LE3 9QP, UK; 5 Leicester British Heart Foundation Centre of Research Excellence, Glenfield Hospital, Leicester LE3 9QP, UK

**Keywords:** atrial fibrillation, procedure time, pulsed-field ablation, radiofrequency ablation, cryoballoon ablation

## Abstract

**Background**: Contemporary AF ablation practice has changed rapidly with the introduction of newer energy-delivery platforms. How these changes translate into longitudinal patterns of technology use and procedural characteristics within routine electrophysiology services is less well described. **Methods**: We performed a retrospective, procedure-level longitudinal service evaluation of AF ablations performed between April 2014 and June 2025 at a United Kingdom (UK) tertiary center. Index and repeat procedures were included. We describe annual technology use and observed skin-to-skin procedure time, fluoroscopy time, and dose-area product (DAP). These are operational measures, not surrogate clinical endpoints. We undertook no adjusted comparative analysis because technology choice was coupled with calendar era and relevant clinical and operator-level covariates were incomplete. **Results**: The analysis included 2862 procedures, comprising 1878 (66%) radiofrequency ablation (RFA), 735 (26%) cryoballoon, and 249 (9%) pulsed field ablation (PFA). RFA remained the predominant approach until 2020, cryoballoon use peaked in 2018 (*n* = 138), and PFA increased from 56 procedures in 2023 to 98 in 2024 and 95 in the first half of 2025. The median skin-to-skin time changed from 205 min in 2014 to 104 min in 2025. Observed median fluoroscopy time changed from 32 to 13 min, and median DAP from 3405 to 782 Gy.cm^2^. **Conclusions**: Over 11 years, the centre experienced a marked change in AF ablation practice, characterised by the progressive adoption of newer technologies, alongside lower recorded procedure durations and radiation exposure. These observations describe service-level temporal trends and should not be interpreted as evidence that any individual ablation modality caused the observed changes or improved clinical outcomes.

## 1. Introduction

Atrial fibrillation (AF) prevalence and incidence have increased substantially during the past two decades and are projected to increase further, creating a major public health challenge worldwide [1]. In conflict-affected settings, AF has also been associated with repeated hospitalisation, new heart failure, and impaired quality of life [2,3,4,5,6,7]. In the United Kingdom (UK), this burden is projected to increase substantially over the coming decades 30 years [1]. Pulmonary vein isolation (PVI) is a cornerstone rhythm-control strategy for symptomatic AF when pharmacological treatment is ineffective or not tolerated.

Catheter ablation technologies have evolved from conventional point-by-point radiofrequency ablation (RFA) to balloon-based cryoballoon ablation and, more recently, non-thermal irreversible electroporation with pulsed-field ablation (PFA). RFA is a mature, versatile approach that can be adapted to complex anatomy and redo procedures, but it requires sequential lesion delivery and carries thermal risks to adjacent structures, thereby driving protective procedural strategies [8]. Cryoballoon ablation provides a single-shot approach to PVI and has shown comparable efficacy and safety to RFA in selected patients with paroxysmal AF, with a simplified and reproducible workflow, although fluoroscopy exposure and phrenic nerve injury remain relevant considerations [9,10,11]. PFA aims to preferentially ablate myocardial tissue while limiting collateral thermal injury. Pivotal and randomised studies have supported its acute efficacy and comparable effectiveness to conventional thermal ablation in selected paroxysmal AF populations, while reporting short procedure durations and a favourable collateral-tissue safety profile [8,12,13,14,15,16,17].

Although trials and contemporary registries have described individual technologies, they do not fully characterise how ablation strategies are adopted and embedded within routine electrophysiology services over time. Longitudinal service evaluations can document the timing of technology adoption and contemporaneous changes in procedural measures. Their role is limited to transparent local audit and hypothesis generation. They do not establish whether a technological change improves clinical effectiveness, procedural safety, patient-centred outcomes, care quality, service capacity, or cost-effectiveness.

Procedural metrics require careful context. Skin-to-skin procedure time captures only the invasive component and can be influenced by venous access, catheter setup, electroanatomical mapping, lesion delivery, adjunctive ablation, anaesthetic workflow, and the experience of the wider laboratory team. Fluoroscopy time and DAP quantify recorded radiation exposure. DAP incorporates exposure intensity and irradiated field size. In this report, these measures are treated as descriptive audit measures, not as measures of procedural efficiency, lesion durability, clinical success, or patient-centred benefit. Differences over time may reflect technology availability, workflow redesign, mapping systems, equipment, case selection, anaesthetic practice, and increasing operator and centre experience.

This retrospective observational study aimed to provide a transparent longitudinal description of AF ablation technology adoption and selected procedural measures over 11 years at a UK tertiary cardiac centre. We assessed changes in the use of RFA, cryoballoon, and PFA, alongside observed skin-to-skin procedure time, fluoroscopy time, and DAP. The study was designed as a single-service audit. It was not designed or powered to evaluate comparative clinical effectiveness, safety, long-term rhythm outcomes, patient-reported outcomes, service capacity, or costs.

## 2. Methods

This retrospective, single-centre, procedure-level observational study included AF ablation procedures performed at a UK tertiary centre between April 2014 and June 2025. The unit of analysis was the procedure, rather than the individual patient. Therefore, patients undergoing repeat ablation could contribute more than one observation. We included index PVI procedures and redo AF ablation procedures. Isolated atrial flutter ablation and accessory pathway ablation were excluded. Procedure and available patient characteristics were extracted retrospectively from electronic hospital systems. AF type was recorded as paroxysmal or non-paroxysmal where available.

The intended procedural endpoint was PVI. The selection of RFA, cryoballoon, or PFA, use of general anaesthesia, mapping system, and any adjunctive ablation strategy were determined by the treating operator, anatomical context, and contemporaneous institutional practice. Detailed lesion-set information, AF duration, and systematic longitudinal clinical follow-up were not uniformly available across the 11-year period. Procedures were performed by consultant electrophysiologists or under the supervision of specialist registrars. Cryoballoon was introduced in 2016, the 90 W/4 s high-power short-duration RF strategy in early 2021, and PFA in 2023. All PFA procedures used the FARAPULSE™ pentaspline large-footprint catheter; no focal PFA catheters were used. Data fields.

### 2.1. Data Collection and Definitions

Data extraction was undertaken from the electronic procedural record and linked hospital systems. Variables were reviewed at the procedural level and included age, sex, AF type where available, index or redo status, primary operator grade, general anaesthesia, ablation modality, and the procedural measures reported in this study. Skin-to-skin procedure time, fluoroscopy time, and DAP were extracted from contemporaneous procedural and radiology records. Each procedure was assigned to the calendar year in which it occurred, and the 2025 data reflect procedures undertaken up to the June 2025 data lock. We used available-case analysis and did not impute missing values. The study period covered multiple technology and documentation eras, and several variables were unavailable or inconsistently recorded in early years. In particular, the dataset did not permit reliable standardisation of patient complexity, systematic adjudication of complications, longitudinal rhythm monitoring, recurrent AF, cardiovascular readmission, patient-reported outcomes, room utilisation, recovery duration, waiting-list measures, or costs. It could therefore not support a credible adjusted comparison of technologies or a retrospective assessment of whether observed procedural trends improved patient care.

The study was reviewed and approved by the University of Leicester ethical committee (reference number: 35479-ia196). Reporting adhered to the Strengthening the Reporting of Observational Studies in Epidemiology (STROBE) guidelines [18].

#### 2.1.1. Peri-Procedural Pathway

Patients were assessed via the Cardiac Rhythm pre-admission pathway prior to elective AF ablation. The pathway included review of the indication for rhythm control, procedural counselling, medication reconciliation, anticoagulation verification, fasting instructions, and assessment of anaesthetic requirements. The choice of RFA, cryoballoon ablation, or PFA was not protocolised. It was determined by the treating electrophysiologist based on AF pattern, prior ablation history, anticipated strategy, pulmonary vein anatomy, where relevant, patient factors, operator preference, and technology availability during the corresponding treatment era.

Adequate oral anticoagulation was an essential component of the pre-procedural pathway. Patients were required to receive oral anticoagulation for at least 4 weeks before ablation unless an alternative strategy had been agreed by the treating team. Patients receiving a direct oral anticoagulant were instructed to take therapy consistently as prescribed. For patients receiving warfarin, anticoagulation control was reviewed through serial international normalised ratio measurements before the procedure. Procedures could be deferred when adequate pre-procedural anticoagulation could not be confirmed. The post-procedural anticoagulation plan was individualised according to thromboembolic risk and clinical indication.

Procedures were undertaken in the cardiac catheter laboratory using femoral venous access, predominantly from the right groin, with contralateral access used when required. Ultrasound guidance for venous access may be used at the operator’s discretion. After vascular access, diagnostic and ablation catheters were advanced under fluoroscopic and, where applicable, electroanatomical mapping guidance. Transseptal puncture was performed to obtain left atrial access, after which intravenous heparin was administered to reduce thromboembolic risk. The procedural endpoint for all three technologies was electrical isolation of the pulmonary veins. If AF persisted after the intended lesion set, direct-current cardioversion could be undertaken at the operator’s discretion.

RFA was performed using a point-by-point technique with three-dimensional electroanatomical mapping. Contact-force sensing was used in most RFA procedures, and high-power, short-duration RFA using the QDOT MICRO™ catheter (Biosense Webster, Inc., Irvine, California, USA) was introduced in early 2021. Cryoballoon ablation used a balloon-based PVI approach, with phrenic nerve monitoring during right-sided pulmonary vein applications. PFA was introduced in 2023. Detailed lesion-set information and energy-delivery parameters were not consistently available across the full 11-year period and were therefore not included in the analyses.

Anaesthetic management was informed by patient factors, procedure type, and contemporaneous local practice. Procedures could be undertaken under conscious sedation, deep sedation, or general anaesthesia. General anaesthesia was used more often in the PFA era. This difference should be considered when interpreting unadjusted procedure time, as anaesthetic workflow and patient movement can independently influence laboratory processes regardless of ablation modality.

#### 2.1.2. Post-Procedural Care and Data Handling

Following ablation, vascular sheaths were removed, and haemostasis was achieved using manual compression and/or a suture-based closure, according to operator practice. Patients underwent bed rest for 2 to 4 h, followed by routine post-procedural observation, including electrocardiography and monitoring of heart rate, blood pressure, and oxygen saturation. Echocardiography was performed before discharge when clinically indicated or in accordance with the contemporaneous local pathway. Patients with an uncomplicated recovery were typically discharged later the same day or the following morning. The current study did not analyse recovery duration, same-day discharge, antiarrhythmic drug management, or post-discharge care pathways because these data were not available consistently throughout the study period.

Data were extracted from the electronic procedural record and radiology system. The procedural record was used for ablation modality, procedure time, anaesthetic approach, repeat-procedure status, and primary operator grade. Fluoroscopy time and DAP were obtained from contemporaneous radiology records. We used available-case analysis for each variable and did not impute missing values. Because the dataset spanned multiple technology, documentation, and workflow eras, denominators vary slightly between variables.

The institutional pathway provides context for the procedural data. Procedure time represents the skin-to-skin invasive component and does not capture pre-procedural preparation, anaesthetic induction, laboratory turnover, recovery, discharge planning, or post-discharge care. Accordingly, a shorter observed procedure duration is not labelled as procedural efficiency in this report and cannot be assumed to translate into greater capacity, lower costs, shorter waiting times, or a better patient experience. Similarly, lower fluoroscopy time and DAP are exposure measures. They should not be considered surrogate measures of lesion durability, clinical success, procedural safety, or patient-centred benefit.

### 2.2. Outcome

The primary descriptive measure was the centre-level annual use of ablation modalities. Secondary descriptive measures were annual skin-to-skin procedure time, fluoroscopy time, and DAP across the 11-year study period. These were operational audit measures and were not used as surrogate endpoints for clinical benefit. Clinical outcomes, including AF recurrence, repeat ablation after the analysed procedure, cardiovascular readmission, antiarrhythmic drug use, patient-reported outcomes, comparative procedural safety, and cost-effectiveness, were not analysed.

### 2.3. Statistical Analysis

Categorical variables are presented as frequency and percentage. Continuous variables are presented as median and interquartile range (IQR). We report descriptive distributions only and do not present formal hypothesis tests or *p*-values for modality or calendar-year comparisons. We did not perform multivariable adjustment, calendar-year adjustment, interrupted or segmented time-series modelling, or formal operator learning-curve analysis. Modality was strongly linked to calendar era, and key case-mix, operator-sequence, workflow, and clinical outcome variables were unavailable or inconsistently recorded. Under these circumstances, statistical testing would imply a comparative precision that the data could not support. Descriptive analyses were performed using GraphPad Prism V10.4 (San Diego, CA, USA).

### 2.4. Analytic Framework and Interpretative Boundaries

The analytic target was the description of longitudinal technology adoption and contemporaneous procedural measures within one service. It was not the causal effect of RFA, cryoballoon ablation, or PFA on procedure time or patient outcomes. Technology availability was closely coupled with treatment era. Incomplete covariate capture precluded meaningful control for case mix, learning curves, and evolving workflows. An adjusted comparative analysis would therefore risk producing spurious technology effects. Findings are reported as service-level temporal observations and should be interpreted as hypothesis-generating only.

## 3. Results

### 3.1. Patient and Procedural Characteristics

A total of 2862 AF ablation procedures were included: 1878 (66%) RFA procedures, 735 (26%) cryoballoon procedures, and 249 (9%) PFA procedures. Because this was a procedure-level analysis, repeated procedures were analysed as distinct observations. Table 1 presents descriptive patient and procedural characteristics together with observed procedural measures. Median age was 62 years, and the cohort was predominantly male. Repeat procedures accounted for 635 procedures (22%), while paroxysmal AF accounted for 1976 procedures (69%). Contact-force sensing was used in 1605 RFA procedures (85%). General anaesthesia was used in 818 procedures (29%), and a consultant was the primary operator in 2575 procedures (90%). The annual proportion of paroxysmal AF procedures was broadly stable, ranging from 67% to 72%. Because treatment selection was non-random and technologies were used in distinct but overlapping calendar eras, the modality columns are descriptive and do not estimate relative performance, safety, effectiveness, or patient benefit.

### 3.2. Temporal Trends in Ablation Modality and Procedural Outcomes

**Figure 1:**
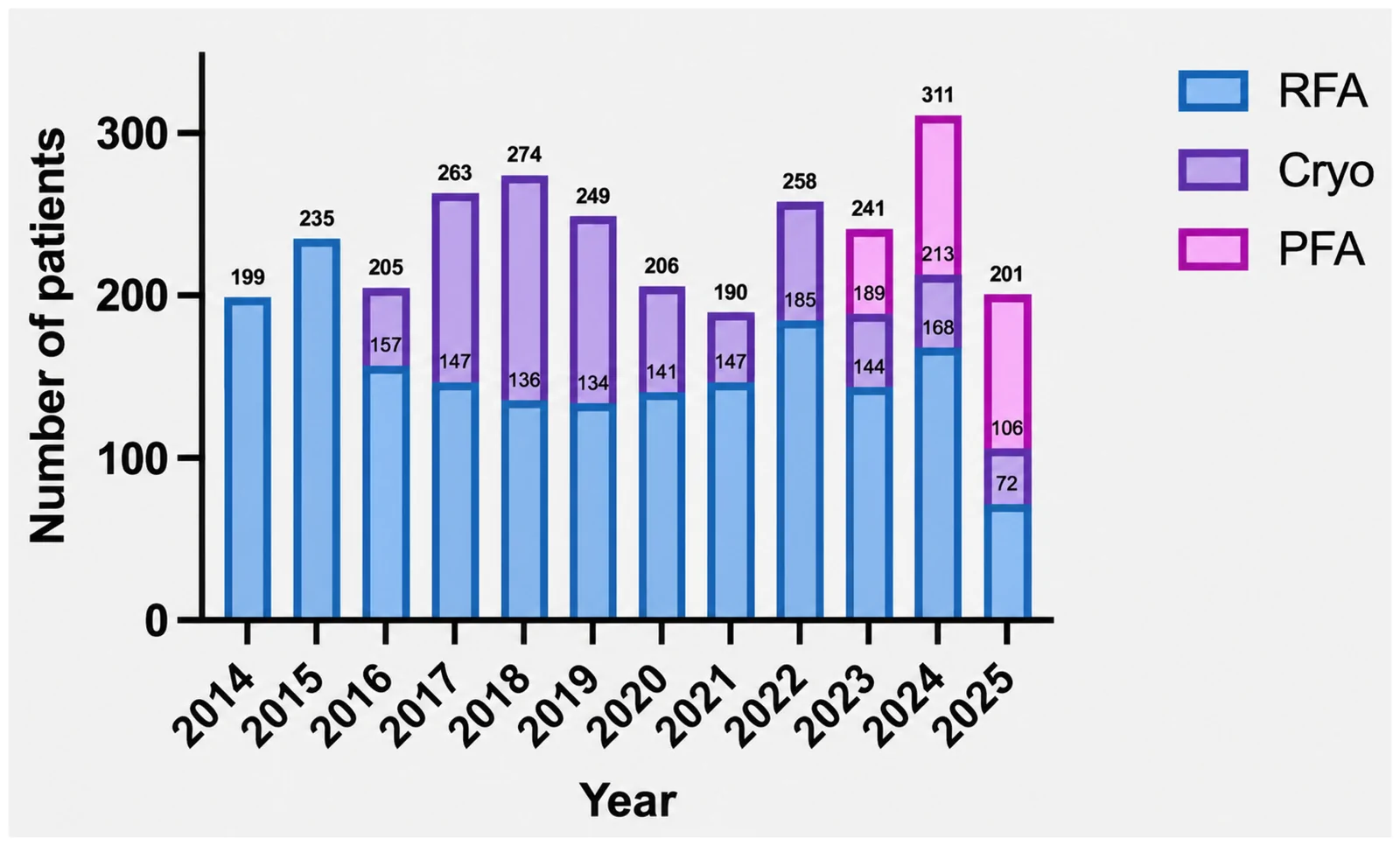
Timeline of different ablation methods used. RF: radiofrequency ablation. Cryo: cryoballoon ablation. PFA: pulsed-field ablation.

Figure 1 shows the annual distribution of ablation modalities. RFA was the predominant modality from 2014 to 2020, peaking at 235 procedures in 2015, then declining to 72 procedures in the first half of 2025. Cryoballoon ablation was introduced in 2016, increased to 138 procedures in 2018, and declined thereafter to 34 procedures in the first half of 2025. PFA was introduced in 2023, with 56 procedures in 2023, 98 in 2024, and 95 in the first half of 2025. These annual PFA counts sum to the reported total of 249 procedures.

**Figure 2:**
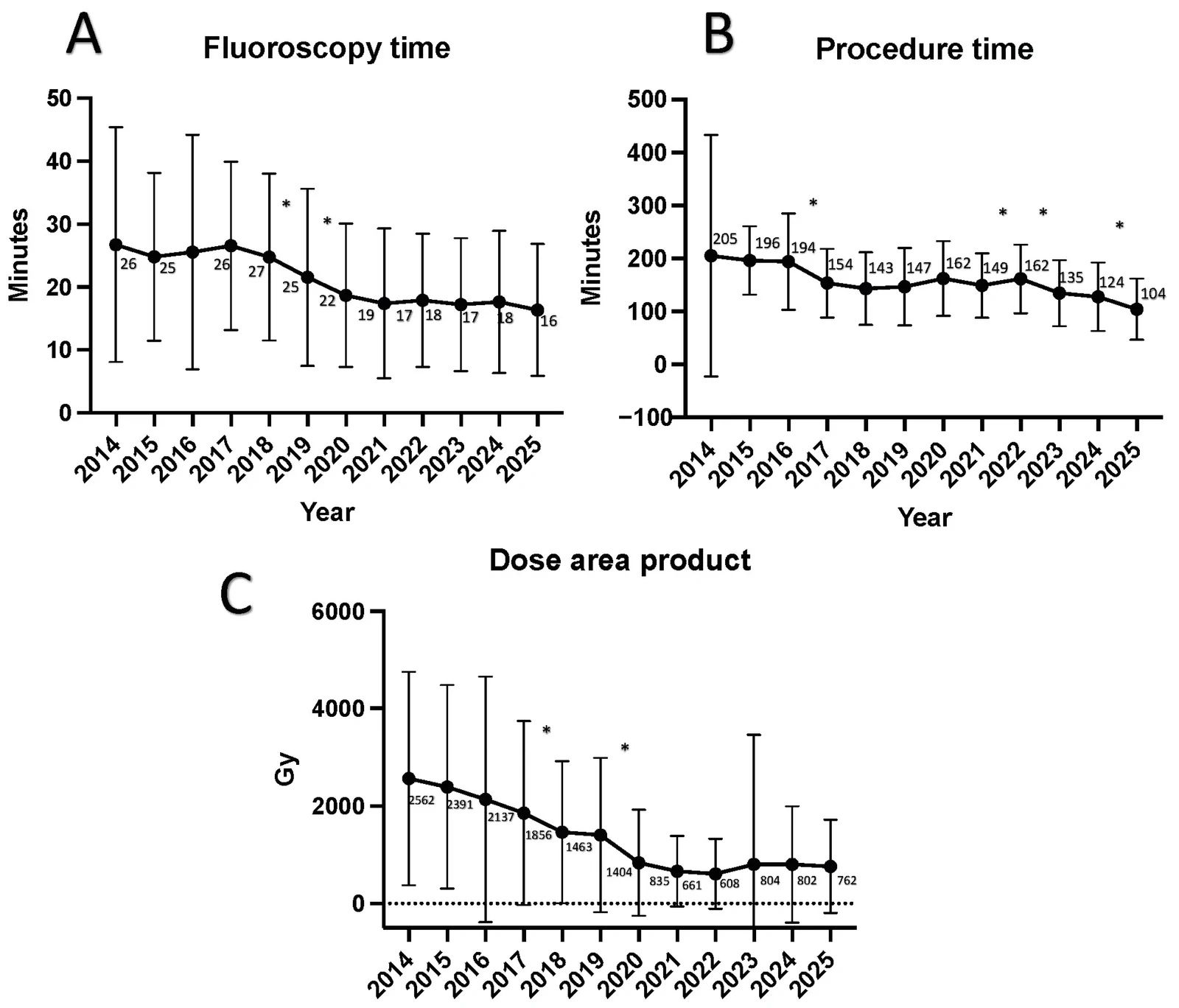
Timeline of fluoroscopy time (**A**), procedure times (**B**), and dose area product (**C**) during the study period. Annual values are shown descriptively and do not represent adjusted technology comparisons. * indicates *p* < 0.05.

Figure 2 summarises annual fluoroscopy time, procedure time, and DAP across the complete procedural cohort. Observed median fluoroscopy time changed from 32 min in 2014 to 13 min in 2025. Observed median skin-to-skin procedure time changed from 205 min in 2014 to 104 min. Observed median DAP changed from 3405 Gy.cm^2^ to 782 Gy.cm^2^. These estimates describe centre-level changes in procedural measures during concurrent evolution in technology and care delivery. They are not adjusted for case mix, calendar year, institutional workflow, or technology adoption and cannot attribute the observed changes to an individual modality.

### 3.3. Within-Modality Descriptive Patterns

**Figure 3:**
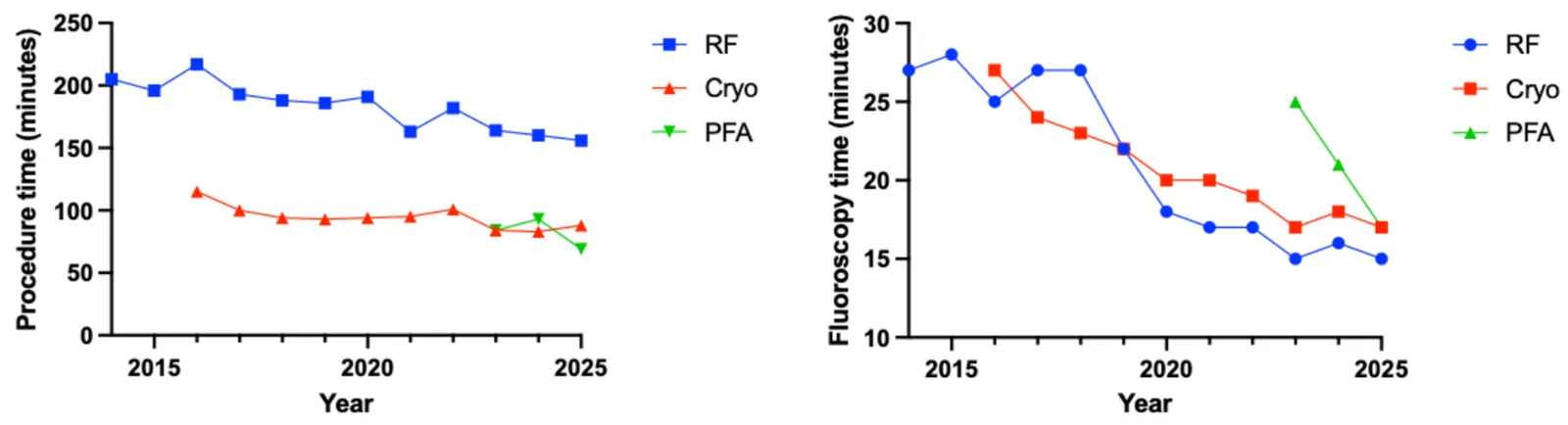
Trends in procedure and fluoroscopy times in the three ablation modalities throughout the study period. RF: radiofrequency ablation. Cryo: cryoballoon ablation. PFA: pulsed-field ablation.

Figure 3 displays within-modality procedure and fluoroscopy patterns to contextualise the broader service evolution. These data are descriptive. Because the modalities were deployed in different eras and the dataset did not account for calendar time, clinical complexity, or learning curves, the results cannot establish a technology-specific effect. In the RFA cohort, observed median procedure time changed from 205 min in 2014 to 156 min in 2025. The corresponding observed medians were 217 min in 2016, 163 min in 2020, and 160 min in 2022.

The 90 W/4 s high-power short-duration (HPSD) strategy using the QDOT Micro™ catheter (Biosense Webster, Inc., Irvine, CA, USA) was introduced at our centre in early 2021. From 2021 to mid-2025, 380 RFA procedures (20.2%) used this approach. In 2024–2025, HPSD was used in 164 of 285 RFA procedures (57.5%).

Within the RFA subgroup, the observed median procedure time was 163 min in 2020 and 156 min in 2025, while the median fluoroscopy time was 17 min in 2020 and 15 min in 2025, respectively. These descriptive observations cannot isolate the effect of the HPSD strategy from other concurrent changes in workflow, technology, or operator experience.

In the cryoballoon subgroup, observed median procedure time was 115 min in 2016, 95 min in 2020, 83 min in 2023, and 88 min in 2025.

In the PFA subgroup, observed median procedure time was 84 min in 2023 and 69 min in 2025.

In the RFA subgroup, observed median fluoroscopy time changed from 27 min in 2014 to 15 min in 2025. In the cryoballoon subgroup, it changed from 27 min in 2016 to 17 min in 2025. In the PFA subgroup, it was 25 min in 2023 and 17 min in 2025. Table 2 presents contemporaneous distributions during the PFA era. These data are descriptive and reflect overlapping but non-random treatment eras.

Procedure-time trends by modality are shown separately in Figure 3. Table 2 is restricted to descriptive contemporaneous fluoroscopy distributions during the PFA era. No formal cross-modality statistical testing was undertaken, and no inference about comparative clinical performance or patient benefit should be drawn from these distributions.

## 4. Discussion

This 11-year, procedure-level service evaluation documents changes in AF ablation technology adoption and selected procedural measures at a high-volume UK tertiary centre. RFA and cryoballoon use changed over time, and PFA was adopted after its introduction in 2023. These changes coincided with shorter observed procedure duration and lower recorded fluoroscopy time and DAP. The principal contribution is a transparent centre-level description of this operational pattern. It does not establish that the changes improved patient care. Differences in treatment era, patient selection, operator and centre experience, mapping technology, anaesthetic practice, and workflow preclude causal attribution to any individual modality. The study did not assess clinical effectiveness, complications, long-term rhythm control, patient-reported outcomes, readmissions, or cost-effectiveness. The findings are hypothesis-generating only.

RFA was the dominant modality in the early study years, consistent with its established role in PVI and its flexibility for complex anatomy and redo procedures. Cryoballoon ablation was introduced in 2016 and reached its highest annual volume in 2018. The decline in cryoballoon use preceded the local adoption of PFA and therefore cannot be attributed solely to PFA replacement. The observed pattern is likely to reflect changing local preferences, case selection, operator experience, and evolving RF workflows, including increasing use of contact-force and ablation-index-guided approaches. However, these factors were not measured systematically and should not be regarded as causal explanations. Randomised data support broadly comparable rhythm-control efficacy of cryoballoon and RFA in selected paroxysmal AF populations [9,11].

**Figure 4:**
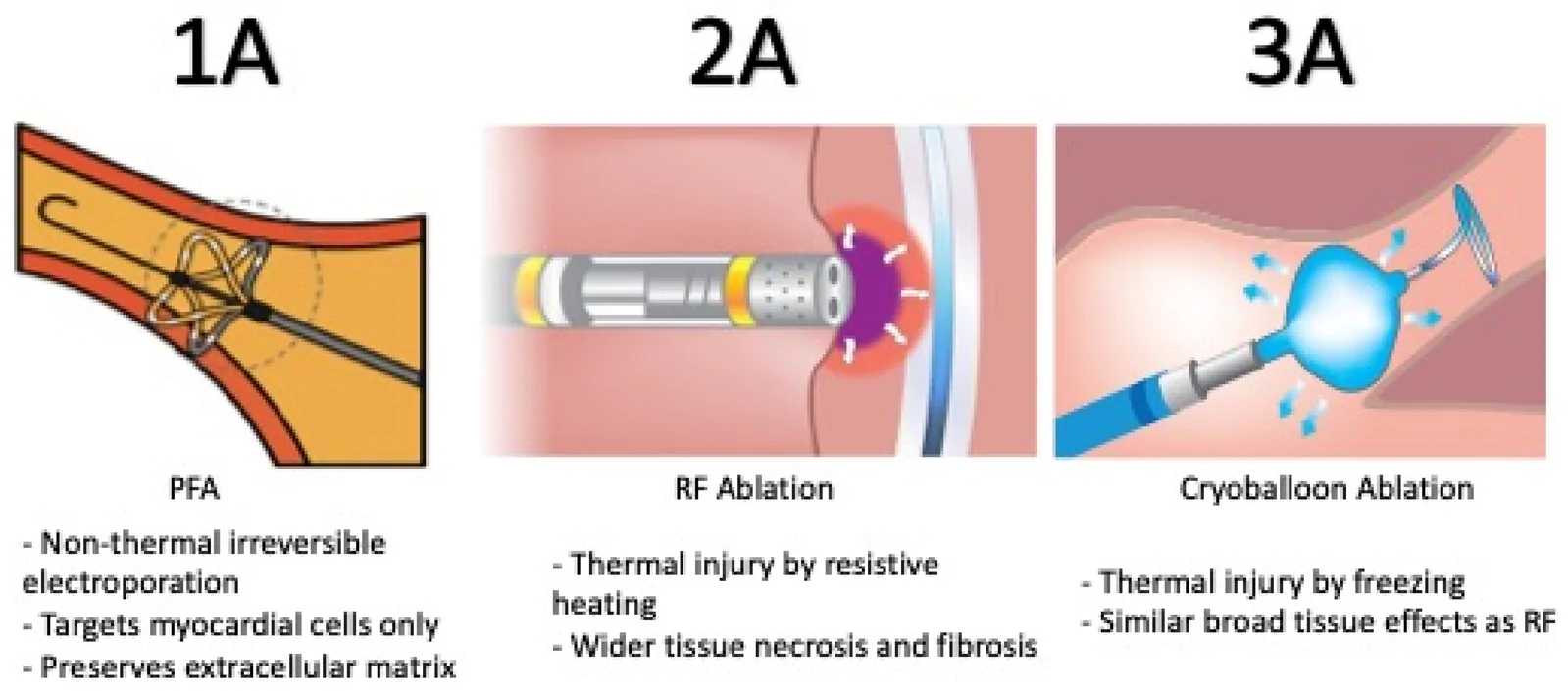
Comparison of pulsed-field ablation (**1A**), radiofrequency ablation (**2A**), and cryoballoon ablation (**3A**) for atrial fibrillation.

PFA is a non-thermal modality based on irreversible electroporation. In this cohort, PFA use increased from 56 procedures in 2023 to 95 procedures in the first half of 2025. Within the complete cohort, PFA procedures had the shortest unadjusted median procedure time and the lowest median DAP. These are era-dependent associations, not comparative results. All PFA procedures were performed during the most recent treatment era, predominantly under general anaesthesia, and without adjustment for case mix, calendar year, operator experience, or institutional workflow. Although pivotal, randomised, and post-approval studies support PFA as an effective alternative to thermal ablation in selected populations [13,14,15,16,17], our data do not establish comparative clinical efficacy, safety, or superiority. In particular, we did not assess oesophageal injury, phrenic nerve injury, stroke or transient ischaemic attack, vascular complications, tamponade, procedure-related mortality, arrhythmia recurrence, repeat ablation, cardiovascular readmission, or patient-reported outcomes. The data therefore cannot show that PFA improved clinical care, recovery, resource use, or costs. Figure 4 illustrates the different energy-delivery concepts.

The findings are suitable only for local procedural audit. They should not be interpreted as evidence that one modality improves clinical outcomes, procedural safety, patient-centred outcomes, capacity, or total service costs. Skin-to-skin procedure time captures only part of the patient pathway. Total laboratory utilisation also depends on the processes of room preparation, anaesthetic induction, vascular access, transseptal puncture, mapping, haemostasis, recovery, and discharge. Similarly, lower fluoroscopy time and DAP are recorded exposure measures. They do not measure lesion durability, freedom from AF, symptom improvement, patient satisfaction, or patient-centred benefit.

The central contribution of this study is a transparent record of technological implementation and observed procedural measures in one tertiary electrophysiology service. Such a longitudinal audit can identify local patterns for quality-improvement projects, radiation-exposure benchmarking, workforce planning, and future data-linkage studies. It cannot substitute for a causal comparative-effectiveness study, and its findings should not be used alone to select an ablation modality for an individual patient.

In this dataset, later calendar years coincide with shorter observed procedural measures. The contribution of ablation technology cannot be separated from increasing operator and team experience, fluoroscopy-sparing workflows, mapping systems, anaesthetic practice, case selection, and other temporal changes. Future prospective studies should collect room-to-room time, recovery duration, same-day discharge, staff requirements, procedural costs, and waiting-list measures. They should link these operational measures to prespecified clinical endpoints, including validated AF recurrence, repeat ablation, complication adjudication, antiarrhythmic drug use, cardiovascular readmission, and patient-reported health status. Such studies could determine whether changes in technology and workflow translate into improved patient care or more effective use of electrophysiology resources.

Our cryoballoon procedure times in the later study years and our early PFA procedure times are broadly within ranges reported in contemporary trials. However, direct numerical comparison is not appropriate because trial populations, protocols, operator experience, and endpoint definitions differ from those in a long-term real-world cohort [11,13,15,16]. Previous centre-level work has reported long-term patient-reported outcomes after RF and cryoballoon ablation, underscoring that shorter observed procedure duration cannot be interpreted as a surrogate for patient benefit (19).

The recorded reduction in fluoroscopy time and DAP is relevant to radiation-exposure audits because lower exposure is desirable for patients and staff. These trends may reflect combined changes in three-dimensional mapping, fluoroscopy-sparing workflows, equipment, procedural planning, team coordination, and operator and centre experience. The present dataset cannot determine whether lower exposure translated into fewer radiation-related harms or other patient benefits. Similarly, shorter observed procedure duration may have implications for laboratory throughput and resource use, but this study did not measure scheduling efficiency, waiting times, recovery time, hospital capacity, or costs. It cannot quantify service-level or patient-level benefit.

The introduction of HPSD RFA coincided with shorter observed RFA procedure times in later years. The observational design cannot separate its contribution from calendar time, learning effects, staffing, mapping systems, or patient complexity. We did not conduct a formal learning-curve analysis because operator-level chronological case sequences and complete covariate data were not available in a form suitable for robust modelling.

General anaesthesia was used more commonly with PFA than with RFA or cryoballoon ablation in this cohort. This may reflect local early-adoption practice rather than an intrinsic requirement of PFA. Hospital stay was short across modalities, but it is a crude process measure and does not establish better recovery or patient-centred benefit. The present data do not include patient discomfort, recovery time, quality of life, symptom status, return to usual activities, or patient satisfaction. These outcomes require prospective patient-centred evaluation.

Future multicentre prospective studies should use harmonised data collection across technology eras. Such work should link procedural measures to prespecified clinical endpoints, including validated definitions of AF recurrence, repeat ablation, complication adjudication, cardiovascular readmission, antiarrhythmic medication use, and patient-reported health status. It should also capture case complexity, left atrial structure and function, AF duration, lesion set, operator case sequence, anaesthetic pathway, room-to-room time, recovery, resource use, and cost. These data would allow adjusted analyses that distinguish technology effects from learning curves and evolving service models. The present findings require external validation and should be considered hypotheses for future evaluation, not conclusions about comparative patient care.

## 5. Limitations

This study has several limitations. It was retrospective, descriptive, single-centre, and procedure-level, which limits external validity. The cohort came from a high-volume tertiary electrophysiology service with local practices, case selection, protocols, and staffing that may not generalise to other centres. The lack of linked clinical outcome data is the most important limitation. The study did not include validated longitudinal measures of AF recurrence, freedom from arrhythmia at 12 months, repeat ablation after the analysed procedure, cardiovascular readmission, antiarrhythmic drug use, or patient-reported outcomes such as AFEQT, EHRA class, EQ-5D, patient satisfaction, or time to return to usual activities. It also did not include complete safety, recovery, capacity, or economic data. Accordingly, the results demonstrate procedural trends only. They cannot establish improved clinical effectiveness, procedural safety, quality of care, patient-centred benefit, service-level benefit, or cost-effectiveness.

Important clinical covariates, including left atrial size, AF duration, CHA_2_DS_2_-VASc score, left ventricular ejection fraction, and major comorbidities, were not consistently recorded across the historical dataset and could not be harmonised for adjustment. Data fields for immediate complications and abandoned procedures were also incomplete and non-standardised. We therefore did not perform or report a comparative safety analysis. The study cannot determine whether evolving technology reduced cardiac tamponade, stroke or transient ischaemic attack, vascular complications, phrenic nerve injury, oesophageal injury, or procedure-related mortality. The availability of technology was closely coupled with calendar time. Comparisons between RFA, cryoballoon, and PFA are further confounded by different treatment eras, progressive workflow and mapping-system changes, and operator and centre learning effects. We did not undertake calendar-year adjustment, segmented temporal analyses, or formal learning-curve modelling because the data required to distinguish these interdependent factors were not available. No causal comparative claims should be drawn from these results. Multicentre studies with standardised patient, procedural, safety, follow-up, and economic data are needed to validate the operational observations.

## 6. Conclusions

Over 11 years, the use of AF ablation technology shifted from a predominant reliance on RFA and cryoballoon to rapid uptake of PFA after 2023. These changes coincided with shorter observed procedure duration and lower recorded fluoroscopy time and DAP. This is a single-centre operational service evaluation. It is hypothesis-generating and does not demonstrate improved procedural efficiency, comparative safety, clinical effectiveness, patient-centred benefit, service-level benefit, cost-effectiveness, or superiority of one ablation modality over another. Prospective multicentre studies with standardised clinical, safety, long-term rhythm, patient-reported, operational, and economic outcomes are required.

## Figures and Tables

**Table 1:** Descriptive patient and procedural characteristics and observed procedural measures by ablation modality.

Variable	Total	RF (*n* = 1878)	Cryo (*n* = 735)	PFA (*n* = 249)
Patient and Procedural Characteristics				
Age (years)	62 (54–69)	62 (54–68)	63 (55–69)	63 (56–70)
Male	1973 (69%)	1326 (71%)	502 (68%)	167 (67%)
Repeat procedure	635 (22%)	581 (31%)	27 (4%)	19 (8%)
Paroxysmal atrial fibrillation ablation	1976 (69%)	1279 (68%)	540 (73%)	162 (65%)
Consultant first operator	2575 (90%)	1755 (93%)	622 (85%)	218 (88%)
General anaesthesia	818 (29%)	506 (27%)	109 (15%)	223 (90%)
Procedural outcomes				
Fluoroscopy time (minutes)	19 (11–28)	18 (10–28)	20 (15–27)	19 (13–27)
Dose area product (Gy.cm^2^)	753 (352–1566)	840 (351–1811)	695 (396–1278)	607 (336–1147)
Skin to skin procedure time (minutes)	142 (94–203)	180 (137–224)	91 (75–111)	73 (52–100)
Hospital stay (days)	1 (0–1)	1 (1–1)	1 (0–1)	0 (0–1)

Data are presented as *n* (%) or median (IQR). Procedure time, fluoroscopy time, DAP, and hospital stay are observed procedural measures, not baseline variables. Available case denominators vary slightly between variables because several historical data fields were incomplete. No between-modality hypothesis testing is reported because technology and treatment era could not be separated in the available data.

**Table 2:** Descriptive fluoroscopy time by ablation modality between 2023 and 2025. No formal cross-modality statistical testing was undertaken.

Year	Radiofrequency	Cryoballoon	Pulsed-Field
2023	13 (7–21)	18 (11–23)	23 (13–19)
2024	15 (7–21)	16 (12–23)	18 (13–28)
2025	11 (7–17)	16 (11–19)	15 (11–24)
Overall	14 (7–20)	16 (11–21)	18 (13–26)

## Data Availability

Data relating to this study are available upon reasonable request from the corresponding author.
